# Endothelial loss during the surgical procedure in saphenous veins harvested by open and endoscopic techniques in coronary artery bypass surgery

**DOI:** 10.17305/bjbms.2020.4656

**Published:** 2020-11

**Authors:** Aleksandra Milutinović, Ruda Zorc-Pleskovič

**Affiliations:** 1 Institute of Histology and Embryology, Faculty of Medicine, University of Ljubljana, Ljubljana, Slovenia; 2 International Center for Cardiovascular Diseases MC Medicor d.d., Izola, Slovenia

**Keywords:** Endothelial cells, saphenous vein, endoscopic harvesting, open harvesting, coronary artery bypass grafting

## Abstract

The patency of the vein graft in coronary artery bypass grafting could be dependent on the conventional open (vsO) or endoscopic (vsE) harvesting and on the hypoxic damage of endothelial cells. We aimed to evaluate both surgical techniques according to endothelial loss that occurs in the time between harvesting and implantation. Twenty-six saphenous veins were divided into vsO (n = 16) and vsE (n = 10) group. Three samples were taken from each vein. The first sample was taken after removal, the second before implantation of the distal part, and the third before the implantation of the proximal part, and they were stained with HE, Movat, and immunohistochemically with CD31. A significant loss of endothelial cells within both groups was found at the time of implantation of the distal and the proximal part of the vein graft compared to the endothelial cells at the time of harvesting. There were no significant differences in the endothelial loss between vsE and vsO groups at the time of harvesting and at the time before the implantation of the distal part. A higher number of endothelial cells was found in vsE group compared to vsO group at the time just before the implantation of the proximal part. The comparison of the implanted portions of vsE and vsO grafts to mammary arteries revealed a significant loss of endothelial cells only in vsO graft. We conclude that, at the time of implantation, the endothelial layer of the vein graft harvested endoscopically is more preserved than of the vein graft harvested openly.

## INTRODUCTION

The leading cause of death worldwide, especially in ­developed countries, is coronary artery disease (CAD) [1-4]. The etiology of CAD is atherosclerosis, which obstructs coronary arteries that supply oxygen and nutrition to the heart muscle [5]. The treatment of CAD depends on the degree of obstruction and is either pharmacological or surgical [5]. The most commonly used surgical technique is coronary artery bypass grafting (CABG) that employs arteries or veins as a graft [5,6], among which the most widely used are the mammary artery and the saphenous vein.

Mammary artery grafts have a higher long-term patency rate compared to saphenous vein grafts. However, adequate lengths of mammary artery grafts are not always available. Contrarily, saphenous vein grafts are easily harvested in the adequate length and are thus more commonly used for CABG [7].

Two surgical techniques are in use for saphenous vein harvesting, i.e., the open and endoscopic approach. In the case of the open approach, the saphenous vein is harvested using a linear incision along the course of the vein under direct vision. To eliminate the need for a long incision, and to reduce the discomfort of the patients and the risk of complications, such as edema, hematoma, cellulitis, wound dehiscence and delayed healing [8], the endoscopic approach was developed [9]. Compared to open harvesting, the endoscopic approach leads to higher patient satisfaction because it reduces a lot of postoperative complications, including postoperative pain, risk of wound infection, and duration of hospital stay [9].

However, previous investigations of two databases, where many patients were enrolled, revealed that the endoscopic technique was associated with an increased long-term mortality rate and a higher rate of vein graft failure [9,10]. Contrarily, other studies reported that the endoscopic technique was not associated with a higher rate of vein graft failure and an increased mortality rate [11-13].

A possible explanation for a higher rate of graft failure when using the endoscopic technique could be endothelial damage caused by greater mechanical stress during the harvesting procedure. The endoscopic and open techniques were therefore compared according to viability and the morphologic damage of saphenous vein endothelium [14,15]. Immediately after harvesting, using either the endoscopic or open technique, it was found that endothelial cells were firmly attached to the tunica intima. The endothelial cells exhibited viability and structural integrity [14]. Grafts harvested by both techniques were also compared immediately after harvesting to detect some endothelial cell markers (i.e., von Willebrand factor [vWF], caveolin, endothelial cadherin, and endothelial nitric oxide synthase [eNOS]). No differences in endothelial damage between the groups were found [14,15].

During the surgical procedure, however, vein grafts are not implanted immediately after the harvesting, but they are stored in hypoxic conditions until the implantation. The damage caused by the synergistic action of mechanical stress and hypoxia may occur.

Our study aimed to evaluate both surgical techniques with respect to endothelial loss that occurs between the time of harvesting and implantation. For the endothelial marker, we used cluster of differentiation-31 (CD31), also known as platelet endothelial cell adhesion molecule 1 (PECAM-1).

## MATERIALS AND METHODS

### Patients

In this preliminary study, 26 patients (22 men and 4 women) aged from 57 to 80 years and undergoing CABG surgery were enrolled.

The national Medical Ethics Committee (MEC 170/07/13, MEC 110/03/16) pre-approved the study and all procedures. Written informed consent was obtained from each patient included in the study. The study protocol conforms to the ethical guidelines of the 1975 Declaration of Helsinki.

### Tissue samples and staining

According to the surgery procedure, saphenous veins were divided into two groups: open (vsO, n = 16) and endoscopic group (vsE, n = 10). In the vsE group, the vein was harvested through a small incision that had been made just below the knee through the subcutaneous tunnel toward the groin area [15,16]. In the vsO group, the saphenous vein harvesting was performed under direct vision through a continuous longitudinal incision in the lower leg following the longitudinal course of the vein [9,15,16].

In all patients, the mammary artery was used as the primary choice for anastomosis for the first vessel grafting, mostly the left anterior descending coronary artery. Just before the implantation of an anastomotic portion, in 9 patients a 0.5 mm long piece was cut from the mammary artery and fixed in formalin (MA group, n = 9).

Three separate 0.5 cm vein samples were taken from each saphenous vein. The first sample was taken from the distal portion of the saphenous vein graft immediately after removal and fixed in formalin.

Next, the venous graft was perfused and stored in a cold (approximately 4°C) heparinized solution (containing 25.000 IU heparin/500 ml saline) until implantation. The second sample was cut just before the implantation of the distal part of the vein graft and the third sample just before the implantation of the proximal part of the vein graft, and they were fixed in formalin.

After 24 hours, the vein samples were dehydrated in alcohol, immersed in xylene, embedded in paraffin, and cut into 4 μm thick transversal step serial sections. The step between the two sections was 50-μm thick. Sections were stored at room temperature and stained with hematoxylin-eosin (HE) and Movat pentachrome [17,18].

Immunohistochemistry was used for the detection of endothelial cells (PECAM-1 or CD31; 1:15, DACO, Glostrup, Denmark), following the manufacturer’s instructions [19].

### Image analysis

Image analysis was performed under a light microscope (Nikon Eclipse E 400, Florida, USA), using a camera (Nikon digital sight DS-M5) and the computer program NIS elements version 3.

The measurements were performed in samples collected just before the implantation in the MA group, and at all 3-time points from harvesting to implantation in the vsO and vsE group.

### Number of endothelial cells/mm

The number of endothelial cells (N), stained immunohistochemically for CD31, was counted at an objective magnification ×40 and expressed as N/mm of the lumen. The lumen of vein and artery samples was manually outlined.

### Statistical analysis

Analyses were performed using IBM SPSS Statistics for Windows, Version 20.0. (IBM Corp., Armonk, NY) and Microsoft Excel 2010. The average values of the measured parameters were calculated for the vsE, vsO, and MA group and expressed as the mean ± SD. The statistical significance of the differences between the vsE and vsO groups was evaluated by the Student’s t-test (*p* < 0.05). The Student’s paired t-test (*p* < 0.05) assessed the statistical significance of the differences between the three venous segments (at acquisition, distal, and proximal parts of the vein graft) within both groups. The statistical significance of the differences between the vsE, vsO, and MA groups was evaluated by analysis of variance (ANOVA) followed by Bonferroni post-hoc test (*p* < 0.05).

## RESULTS

### Patients

The study enrolled 26 patients. Depending on the surgical method of vein harvesting, they were divided into two groups: vsE (n = 10) and vsO (n = 16). In both groups, there were more men than women (Table 1). All patients had hypertension. There were no significant differences in age, blood pressure, glucose levels, total cholesterol, low-density lipoprotein (LDL), high-density lipoprotein (HDL), triglyceride levels, creatinine, and urea in plasma between groups (Table 1).

**TABLE 1:**
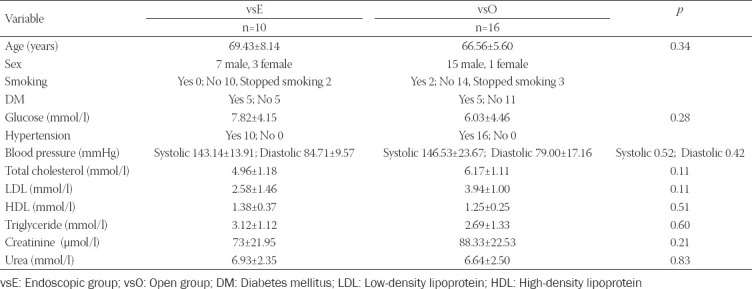
Characteristics of patients in the vsE and vsO group

### Outcome

During the period of 1 to 2 years after the surgical procedure, all patients felt well. One patient from the vsO group developed wound dehiscence on the leg as a postoperative complication and was re-operated (surgically treated) 1 month after CABG.

### Histological description

**FIGURE 1:**
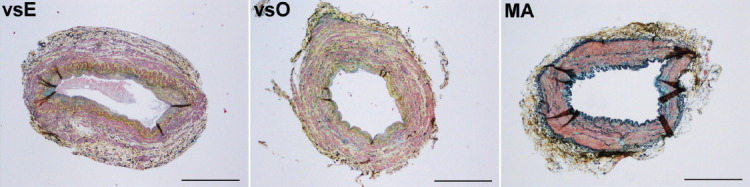
Vein and mammary artery samples stained with Movat pentachrome (collagen fibers in yellow, mucin in blue-green, elastic fibers and nuclei in black, muscle in dark red, and fibrin in bright red color). The tunica intima of venous and arterial samples was composed of the endothelial and the subendothelial layer. Subendothelial layer in the endoscopic (vsE) and open harvested saphenous vein (vsO) samples was thick, composed of connective tissue and smooth muscle cells (SMCs). The tunica intima of the mammary artery (MA) group was very thin. Between the tunica intima and tunica media was an internal elastic lamina that was frequently discontinuous and irregular in the vsE and vsO group and embedded in collagen fibers, and in the MA group it was continuous and prominent. In the vsE and vsO group, the tunica media consisted of longitudinal bundles of SMCs surrounded by a thick layer of circularly oriented SMCs. The layer of longitudinally orientated SMCs in the vsO group was sometimes missing, but in the vsE group often surrounded the whole vascular wall. Within the SMCs there were distinctive bundles of collagen and elastic fibers. The tunica media of the MA group consisted of well visible circularly orientated bundles of SMCs and elastic fibers, surrounded by a distinctive external elastic lamina. In the vsE and vsO group, the tunica adventitia consisted of collagen and elastic fibers and bundles of longitudinally orientated SMCs. The latter was missing in the MA group (bars: vsE, vsO = 1mm, MA = 500 µm).

The histological analysis of veins, stained with HE and Movat pentachrome (Figure 1), showed that the tunica intima was covered with endothelial cells. The subendothelial layer was composed of connective tissue with a lot of ground substance and smooth muscle cells (SMCs). In some segments, it was thickened. Between the tunica intima and tunica media, there was the internal elastic lamina, which was frequently irregular and discontinuous and embedded in the thick layer of collagen fibers. In some places, the internal elastic lamina split into two membranes. Below was a layer of longitudinal bundles of SMCs surrounded by a thick layer of circularly oriented SMCs. The internal elastic membranes mostly surrounded the longitudinally running bundles of SMCs. In the vsO group, the layer of longitudinal SMCs was sometimes missing. In the vsE group, the layer of longitudinal SMCs often surrounded the whole vascular wall. There were elastic fibers embedded in the bundles of collagen fibers within the SMCs, and the adventitial layer with collagen and elastic fibers and bundles of longitudinal SMCs surrounded the media.

The intima of the mammary arteries was mostly thin, surrounded by a prominent internal elastic lamina. The media consisted of a distinctive layer of SMCs that included elastic fibers. Bundles of elastic fibers surrounded the media as an external elastic lamina. The adventitia consisted of collagen and elastic fibers.

### The time points for taking the first, second, and third sample of vein grafts

The time of harvesting was zero when the first samples were taken. The second sample was cut at the time just before the implantation of the distal portion of the vein graft, and the third sample at the time just before the implantation of the proximal part. There were no significant differences in the times of implantation of distal (vsE: 51.50 min ± 19.01; vsO: 72.09 min ± 44.20, *p* = 0.187) and proximal segments (vsE: 108.50 min ± 33.59; vsO: 112.50 min ± 59.04, *p* = 0.848) of grafts between the vsE and vsO group.

### Number of endothelial cells/mm in the vsE and vsO group at the time of harvesting and during the time of implantation of the distal and proximal part

**FIGURE 2:**
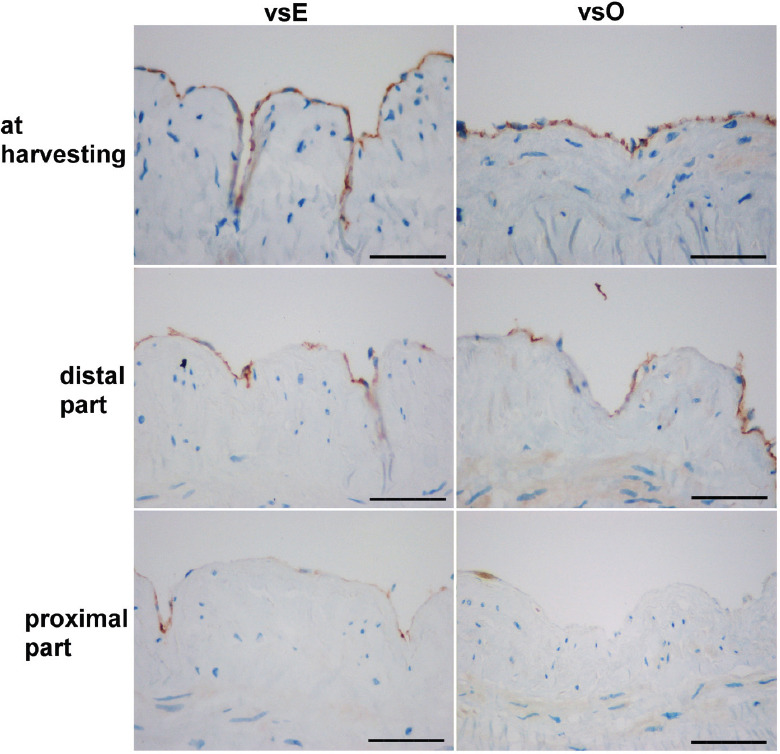
Veins stained for CD31 from the vsE and vsO group at the time of harvesting, and at the time of distal and proximal part implantation (bars = 50 µm). vsE: Endoscopic group; vsO: Open group.

The number of CD31 positive cells (Figure 2) that surround the lumen of each vessel was counted at an objective magnification ×40 and expressed as N/mm of the lumen.

**FIGURE 3:**
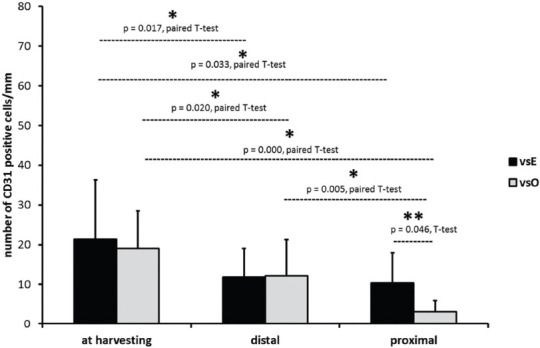
The average number of CD31 positive cells at the time of harvesting and just before the implantation of the distal part and the proximal part of the vein graft. Note that a higher number of CD31 positive cells was found in the vsE group compared to the vsO group just at the time of the proximal part implantation (**significantly different number of CD31 positive cells between the vsE and vsO group, Student’s t-test). Within the vsE group, there were significantly fewer CD31 positive cells at the time when the distal and proximal portions were implanted, compared to the number of CD31 positive cells at the time of harvesting. Note that within the vsO group, the results were similar but more severe. (*significantly different number of CD31 positive cells between time points within the vsE and vsO group, paired Student’s t-test). vsE: Enoscopic group; vsO: Open group.

There were no significant differences in the average number of CD31 positive cells between the two groups at the time of harvesting (vsE: 22.56 ± 12.36; vsO: 19.04 ± 9.44; *p* = 0.419) and just before the implantation of the distal part (vsE: 11.83 ± 7.32; vsO: 12.18 ± 9.11; *p* = 0.920) (Figure 3). Just before the implantation of the proximal part, we observed a higher number of CD31 positive cells in the vsE group compared to the vsO group (vsE: 10.35 ± 7.62; vsO: 3.14 ± 2.75; *p* = 0.016; Figure 3).

Within the vsE group, there were significantly fewer CD31 positive cells at the time of the distal and proximal portion implantation (*p* = 0.026; *p* = 0.027, respectively, paired Student’s t-test) compared to the number of CD31 positive cells at the time of harvesting (Figure 3). We found no difference in the number of CD31 positive cells between the proximal and distal segments (Figure 3).

The results were similar but more severe within the vsO group. There were significantly fewer CD31 positive cells at the time when the distal and proximal parts were implanted (*p* = 0.020; *p* = 0.000, respectively, paired Student’s t-test) compared to the number of CD31 positive cells at the time of harvesting. There were also differences in the number of CD31 positive cells between the distal and proximal parts (*p* = 0.005; Figure 3).

### Number of endothelial cells/mm in implanted segments in the vsE, vsO, and MA group

**FIGURE 4:**
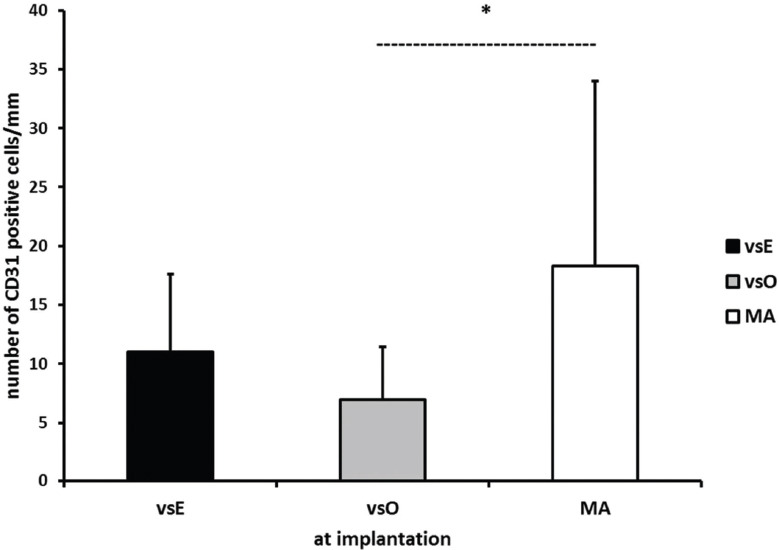
The average number of CD31 positive cells at the time of implantation in the vsE, vsO, and MA group. (*significantly different number of CD31 positive cells between the vsO and MA group ANOVA, Bonferroni post-hoc test). vsE: Endoscopic group; vsO: Open group; MA: Mammary artery group.

We compared the average number of CD31 positive cells between the implanted segments of the venous grafts (the average number from the distal and proximal part) and the implanted arterial segments that served as control. We found significantly fewer endothelial cells in the vsO group (6.85 ± 4.66) compared to the MA group (18.30 ± 15.69, *p* = 0.019 ANOVA, Bonferroni post-hoc test) but not compared to the vsE group (11.03 ± 6.26). We found no significant difference between the vsE and MA group (Figure 4).

## DISCUSSION

This study focused on the differences between the endoscopic and open surgical techniques with respect to endothelial loss that occurs in the period from harvesting to implantation.

We found no differences in the number of CD31 positive cells at the time of harvesting and at the time of implantation of the distal part between the vsE and vsO techniques. However, at the time of implantation of the proximal part, we observed a significant loss of endothelial cells in the veins harvested with the vsO compared to the vsE technique. Previous studies investigated the effect of endoscopic and open saphenous vein harvesting on saphenous vein endothelium only at the time of harvesting [14,15,20]. They observed no significant histomorphological differences in the endothelium between the two groups, such as gross breaks, stretching, or detachment [14,15], which is consistent with our results. A more detailed investigation revealed that the endothelial cell membrane was more damaged in the veins harvested with the endoscopic method [20]. They found functional alterations of endothelial cells such as the altered distribution of eNOS and caveolin [20]. In the veins harvested with the open techniques, they found increased esterase activity, nitric oxide production, and calcium mobilization [20]. Further investigations did not find differences in caveolin, eNOS, vWF, and cadherin expression in endothelial cells between the two groups [14,15]. They found only increased calcium mobilization and increased nitric oxide production in endothelial cells after Bradykinin stimulation in the vsO compared to the vsE group [14].

In this study, we also did not find differences in endothelial loss between the vsE and vsO groups at the time of implantation of the distal part of the vein graft. The distal portions of vein grafts harvested with the vsE method were implanted 52 min ± 19 after harvesting and with the vsO method 72 min ± 44 after harvesting; the time of implantation was not significantly different between the two groups. During this time, the venous grafts were stored in a cold heparinized solution. There was a significant loss of endothelial cells at the time of implantation of the distal part in both groups compared to the portion of the vein graft at the time of harvesting, suggesting adverse hypoxic conditions in the heparinized solution even though the solution was cooled, which reduced metabolism and consequent oxygen demand [21].

At the time of implantation of the proximal part, we observed a significant loss of endothelial cells in the veins harvested with the open compared to the endoscopic technique. The time of implantation was statistically not different; the proximal parts were implanted 57 min and 41 min after the implantation of the distal portion in the vsE and vsO group, respectively. A significant loss of endothelial cells at the time of implantation of the proximal part compared to the distal part was observed in the vsO but not in the vsE group.

Moreover, the comparison of the implanted segments between the vsE and vsO vein grafts and MAs revealed a significant loss of endothelial cells in the vsO but not in the vsE group compared to the MA group. Unexpectedly, our results showed that the vsO technique is better than the vsE technique. According to the previous research [14,15,20], we expected equal or more significant loss of endothelial cells in the vsE than in the vsO group as the endoscopic method causes more mechanical stress to the vessels than the open method. This result may be due to the small number of veins, especially in the vsE group – which is the main weakness of our study, and/or due to some unknown mechanisms. Previously, it was shown that the integrity of the endothelium is affected by distension, temperature, the composition of storage solutions, and pH [20]. The effects of these factors can be synergistic and may occur later. However, according to our results, the difference between the groups occurred during the time between the implantation of the distal and proximal ends. During this time, both groups experienced the loss of endothelial cells. The mammary artery is known to be best suited for grafts because they remain transient, as atherosclerosis almost does not occur. However, the analysis of endothelial loss at the time of implantation in the vsE and vsO groups compared to the MA group showed endothelial loss only in the vsO group.

The endothelial lining serves as a physiological barrier between the subendothelial layer and blood [2]. Injury of the endothelium during surgical manipulation can form an initiation site for the formation of late-stage atheromas and, consequently, graft failure [20]. It was also reported that endothelial injury of the vein graft could lead to a decrease in the antithrombogenic properties, vasospasm, thrombogenesis, occlusive intimal hyperplasia, and stenosis [20].

Nevertheless, 1 to 2 years after surgery, none of our patients showed clinical signs of arterial or venous graft failure following the endoscopic or open harvesting. It seems that endothelial damage caused by a single short-term manipulation does not necessarily have a severe impact on the development of atherosclerosis. It is known that atherosclerosis does not always occur at the endarterectomy site, where the entire intima and media are removed. Our results showed that the loss of endothelial cells during any surgical procedure is not necessarily relevant for the outcome of the graft.

## CONCLUSION

We conclude that, at the time of implantation, the endothelial layer of saphenous veins harvested endoscopically is more preserved than the endothelium of saphenous veins harvested using the open method. However, none of our patients showed clinical signs of arterial or venous graft failure following the endoscopic or open harvesting 1 to 2 years after surgery.
